# Involvement of the YneS/YgiH and PlsX proteins in phospholipid biosynthesis in both *Bacillus subtilis *and *Escherichia coli*

**DOI:** 10.1186/1471-2180-7-69

**Published:** 2007-07-24

**Authors:** Mika Yoshimura, Taku Oshima, Naotake Ogasawara

**Affiliations:** 1 Graduate School of Information Science, Nara Institute of Science and Technology, Ikoma, Nara 630-0192, Japan

## Abstract

**Background:**

Phospholipid biosynthesis commences with the acylation of glycerol-3-phosphate (G3P) to form 1-acyl-G3P. This step is catalyzed by the PlsB protein in *Escherichia coli*. The gene encoding this protein has not been identified, however, in the majority of bacterial genome sequences, including that of *Bacillus subtilis*. Recently, a new two-step pathway catalyzed by PlsX and PlsY proteins for the initiation of phospholipid formation in *Streptococcus pneumoniae *has been reported.

**Results:**

In *B. subtilis*, 271 genes have been reported to be indispensable, when inactivated singly, for growth in LB medium. Among these, 11 genes encode proteins with unknown functions. As part of a genetic study to identify the functions of these genes, we show here that the *B. subtilis *ortholog of *S. pneumoniae *PlsY, YneS, is required for G3P acyltransferase activity, together with PlsX. The *B. subtilis *genome lacks *plsB*, and we show in vivo that the PlsX/Y pathway is indeed essential for the growth of bacteria lacking *plsB*. Interestingly, in addition to *plsB*, *E. coli *possesses *plsX *and the *plsY *ortholog, *ygiH*. We therefore explored the functional relationship between PlsB, PlsX and YgiH in *E. coli*, and found that *plsB *is essential for *E. coli *growth, indicating that PlsB plays an important role in 1-acyl-G3P synthesis in *E. coli*. We also found, however, that the simultaneous inactivation of *plsX *and *ygiH *was impossible, revealing important roles for PlsX and YgiH in *E. coli *growth.

**Conclusion:**

Both *plsX *and *yneS *are essential for 1-acyl-G3P synthesis in *B. subtilis*, in agreement with recent reports on their biochemical functions. In *E. coli*, PlsB plays a principal role in 1-acyl-G3P synthesis and is also essential for bacterial growth. PlsX and YgiH also, however, play important roles in *E. coli *growth, possibly by regulating the intracellular concentration of acyl-ACP. These proteins are therefore important targets for development of new antibacterial agents.

## Background

**Figure 1:**
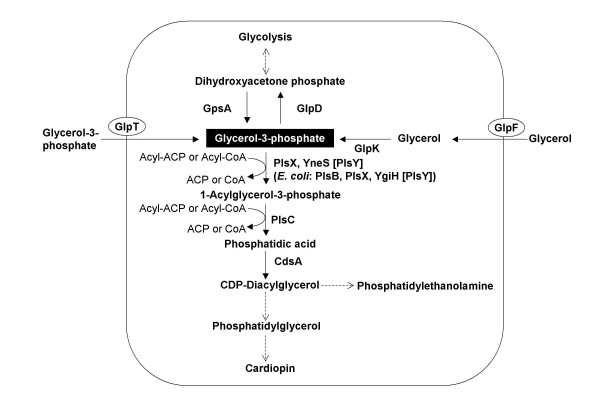
**Phospholipid synthesis pathway in *B. subtilis***. Abbreviations: GpsA, NAD(P)H-dependent glycerol-3-phosphate dehydrogenase; GlpD, glycerol-3-phosphate dehydrogenase; GlpT, glycerol-3-phosphate permease; GlpF, glycerol uptake facilitator protein; GlpK, glycerol kinase; PlsC, 1-acylglycerol-3-phosphate O-acyltransferase; CdsA, phosphatidate cytidylyltransferase.

Phospholipids are major components of the cell membrane. Glycerol-3-phosphate (G3P) forms the backbone of all phospholipid molecules [1]. Phospholipid biosynthesis begins with two steps of G3P acylation, leading to the formation of phosphatidic acid (PA). In bacteria, PA is converted to CDP-diacylglycerol, the precursor of the three major phospholipids, phosphatidylethanolamine (PE), phosphatidylglycerol (PG), and cardiolipin (CA) [2] (Figure 1). Earlier biochemical and genetic analyses disclosed that G3P and 1-acylglycerol-phosphate (1-acyl-G3P) acyltransferase are encoded by the *plsB *and *plsC *genes, respectively, in *Escherichia coli *[3-5]. Interestingly, while *plsC *is universally conserved in eubacteria, *plsB *has been identified in only a limited number of species, mainly belonging to the gamma proteobacterial group. Another gene homologous to *E. coli plsC*, possibly encoding G3P acyltransferase, *plsD*, has been cloned from the *Clostridium butyricum *genome, based on its ability to complement *plsB *defects in *E. coli*. However, the role of *plsD *in *C. butyricum *cells is currently unclear [6].

*E. coli *PlsB utilizes acyl-ACP (Acyl Carrier Protein) and acyl-CoA as acyl donors to synthesize 1-acyl-G3P [7]. Recently, Lu et al. [8,9] have reported a new two-step pathway that utilizes a novel fatty acid intermediate for the initiation of phospholipid formation in *Streptococcus pneumoniae*. They demonstrated biochemically that PlsX produced a unique activated fatty acid by catalyzing the synthesis of fatty acyl-phosphate from acyl-ACP, and then showed that PlsY transferred the fatty acid moiety from acyl-phosphate (acyl-PO_4_) to G3P. The *plsX *gene is widely conserved in eubacteria and it had been suggested that this gene was involved in fatty acid and/or phospholipid synthesis in *E. coli*, although the exact role of the gene remained unknown [3]. In *B. subtilis*, PlsX is essential for growth [10] and PlsX expression is controlled by FapR, a protein that regulates fatty acid and phospholipid biosynthesis genes [11]. Although the *plsY *gene, encoding a membrane protein around 200 amino acids long, is also widely conserved in eubacteria [8,9], the *plsY *gene function had been completely unknown.

In the gram-positive spore-forming bacterium,*Bacillus subtilis*, we showed that 271 genes, including *plsC *(*yhdO*) and *plsX*, when inactivated singly, were indispensable for growth in LB medium [10]. Among these genes, 11 encoded proteins with unknown functions, and these proteins are therefore targets for development of new antibacterial agents. As part of a genetic study to identify the functions of these genes, here we show that the *B. subtilis *ortholog of *S. pneumoniae *PlsY, YneS, is required for G3P acyltransferase activity, together with PlsX. The *B. subtilis *genome lacks *plsB*, and our results demonstrate in vivo that the PlsX/Y pathway is essential for the growth of bacteria lacking *plsB*. Interestingly, in addition to *plsB*, *E. coli *possesses *plsX *and the *plsY *ortholog, *ygiH*. Thus, we explored the functional relationship between PlsB, PlsX and YgiH in *E. coli*, and found that, although PlsB plays the principal role in 1-acyl-G3P synthesis, PlsX and YgiH are also important for optimal *E. coli *growth.

## Results

### Isolation of a *B. subtilis yneS-ts *mutant

To explore the functions of essential genes of unknown function, we adopted a strategy to isolate temperature-sensitive (*ts*) mutants of these genes and then to seek extragenic suppressors of the *ts *genes. To generate a *ts *mutation in *yneS*, we initially introduced a nonfunctional chloramphenicol-resistant gene (*cat*) downstream of *yneS*, and transformed cells with the *yneS*-functional *cat *fragment mutagenized by PCR in vitro. Chloramphenicol-resistant transformants were selected at 30°C, and colonies that were unable to grow above 42°C were analyzed. In this manner, we isolated a *ts *mutant of *yneS *(strain MY103) that had a one-base substitution altering Val190 to Glu in the C-terminal cytoplasmic region of the protein.

### Mapping of the suppressor mutation site in the *yneS-ts sup-1 *strain

**Figure 2:**
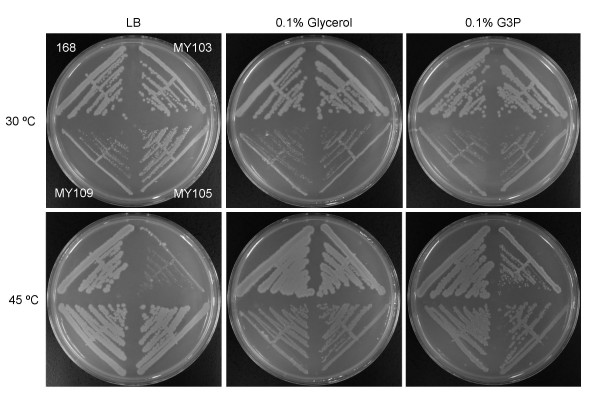
**Growth properties of *B. subtilis yneS-ts *and suppressor mutants**. *B. subtilis *wild-type (168), *yneS-ts *(strain MY103), *yneS-ts sup-1 *(strain MY105), and *yneS-ts *Δ*glpD*::pMutinNC (strain MY109) cells were grown in LB, LB with 0.1% (v/v) glycerol, and LB with 0.1% (w/v) glycerol-3-phosphate, at 30°C and 45°C.

Next, we isolated a spontaneous extragenic suppressor mutant of the *ts *phenotype (strain MY105, *yneS-ts sup-1*, Figure 2) by cultivation at the restrictive temperature. Transformation of wild-type strain 168 cells with chromosomal DNA of MY105 led to the *ts *growth of more than 90% of chloramphenicol-resistant transformants, indicating that the *sup-1 *mutation was not linked to the *yneS *gene. MY105 cells lost sporulation ability, and the Spo^- ^phenotype was maintained in MY107 cells (Pspac::*yneS sup-1*) in which the *yneS-ts *gene was replaced with wild-type *yneS *under control of the IPTG-dependent Pspac promoter. A DNA fragment (ca. 8 kb) that complemented the Spo^- ^phenotype of MY107 in trans was isolated from a *B. subtilis *genomic DNA library in which an *Mbo*I partial digest was ligated with the phage vector, φCM, as described previously [12]. The 8 kb fragment was cut into 2.5 kb and 5 kb fragments. The 5 kb fragment with the *cat *gene of the phage vector was cloned into pBR322, while the 2.5 kb fragment was ligated into pCA191 containing the *cat *gene [13]. MY107 cells were transformed with these plasmids for integration into the chromosome via single crossovers. The results indicated that the 5 kb fragment allowed recovery of the ability to sporulate, while the 2.5 kb fragment did not. Sequencing of the 5 kb fragment revealed three genes. These were the 3'-terminal region of *glpF*, encoding the glycerol uptake facilitator, and full-length *glpK *and *glpD*, encoding glycerol kinase and glycerol-3-phosphate dehydrogenase, respectively. Because *glpF *and *glpK *constitute an operon, and because the cloned 8 kb fragment did not contain the promoter sequence [14], *glpK *was possibly not expressed from the fragment cloned in φCM. The *glpD *promoter between *glpK *and *glpD *has, however, been identified [15]. These results suggested that the mutation(s) responsible for the Spo- phenotype mapped in the *glpD *gene. To confirm this theory, full-length wild type *glpD *(in pCA191*glpD*-FL), the 5'-terminal half of the gene (in pCA191*glpD*-N), and the 3'-terminal half (in pCA191*glpD*-C), were separately cloned into pCA191. The resulting constructs were integrated into the MY107 chromosome by single crossovers and the sporulation abilities of transformants were examined. The 5'-terminal region of *glpD*, and the full-length gene, conveyed the ability to sporulate, but the 3'-terminal *glpD *region did not. Sequencing of the 1.2 kb 5'-terminal half of *glpD *in MY103 (*yneS-ts*) revealed a single base (G) insertion between nucleotides C 492 and G 493, which resulted in inactivation of the gene. This result was consistent with the previous finding that *glpD *inactivation impairs sporulation ability [16].

We further confirmed that *glpD *inactivation suppressed the *yneS-ts *phenotype by insertion of a plasmid, pMutinNC [17], carrying an internal segment of the *glpD *gene, into the *glpD *gene of *yneS-ts *cells (strain MY109, *yneS-ts*, Δ*glpD*::pMutin NC, Figure 2).

### Increases in intracellular G3P concentration suppress the *yneS-ts *phenotype

The intracellular G3P concentration is known to be elevated in *glpD *mutants [16]. We speculated that increases in the G3P level might suppress the *yneS*-*ts *phenotype. Indeed, we found that supplementation of the growth medium (LB) with 0.1% (w/v) G3P, or glycerol, complemented the *yneS-ts *phenotype (Figure 2). Growth of MY105 (*yneS-ts sup-1*) and MY109 (*yneS-ts*, Δ*glpD*::pMutinNC) cells was impaired compared to *glpD*^+ ^cells (wild type and *yneS-ts *cells), when glycerol or G3P was supplemented in the growth medium. This phenotype was also observed in MY108 (Δ*glpD*::pMutinNC) cells (data not shown). Given that accumulation of G3P is known to result in abnormal septation and inhibition of sporulation [18,19], an increase in the intracellular concentration of G3P would explain the growth impairment of cells without *glpD*. It should be noted that G3P and glycerol did not complement the growth defect resulting from YneS depletion in the IPTG-dependent *yneS *mutant cells, strain YNESp [10] (data not shown). The YneS-ts mutant protein will retain a reduced activity at high temperature, although the activity is insufficient to support cell growth. Residual activity would be enhanced by increases in the intracellular concentrations of G3P or glycerol.

### Phospholipid synthesis in *B. subtilis plsC*-*ts*, *yneS-ts*, and IPTG-dependent *plsX *mutants, under restrictive conditions

G3P is a substrate for G3P acyltransferase, and inhibition of G3P acyltransferase activity as a result of simultaneous mutations in *plsB *and *plsX *in *E. coli *led to G3P auxotrophy [3]. Accordingly, we examined the involvement of YneS in 1-acyl-G3P synthesis by analyzing phospholipid synthesis in *yneS-ts *cells at the restrictive temperature. We additionally constructed mutations in genes possibly involved in phospholipid synthesis. These included a *ts *mutation in *plsC*, in which 7 C-terminal residues were deleted (strain MY112), and an IPTG-dependent *plsX *mutation in strain MY111.

**Figure 3:**
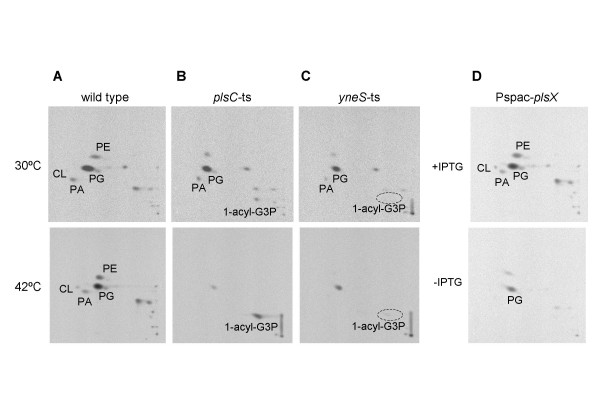
**Phospholipid synthesis in *B. subtilis yneS-ts*, *plsC*-*ts*, and IPTG-dependent *plsX *mutants, under restrictive conditions**. Labeling with ^32 ^[Pi] for 5 min, lipid isolation, lipid separation using two-dimensional TLC, and phospholipid detection, are described in Methods. (A) Wild-type cells labeled at 30°C and 42°C. (B) *plsC*-*ts *cells (strain MY112) labeled at 30°C and 42°C. (C) *yneS-ts *cells (strain MY103) labeled at 30°C and 42°C. (D) Pspac-*plsX *cells (strain MY111) labeled in the presence and absence of IPTG. Abbreviations: 1-acyl-G3P, 1-acylglycerol-phosphate; PA, phosphatidic acid; PE, phosphatidylethanolamine; PG, phosphatidylglycerol; CA, cardiolipin.

To determine phospholipid biosynthesis rates, *B. subtilis *cells were pulse-labeled with ^32 ^[Pi] for 5 min. Phospholipids were extracted using the method of Bligh and Dyer [20], and separated by two-dimensional thin layer chromatography [5]. We detected PA, PG, PE, and CL in wild-type cells growing at either 30°C or 42°C (Figure 3A). When *plsC*-*ts *cells were labeled at 30°C, a pattern similar to that of wild-type cells was obtained. At the restrictive temperature, however, 1-acyl-G3P accumulation was observed, and phospholipid synthesis did not take place (Figure 3B). This is consistent with the known 1-acyl-G3P acyltransferase activity of PlsC. In contract, no phospholipids were detectable in *yneS-ts *cells following a shift to the restrictive temperature (Figure 3C). Our data indicate that YneS inactivation results in blockage of the first step in phospholipid synthesis in *B. subtilis *cells. Furthermore, PlsX depletion in IPTG-dependent mutant cells induces a similar change in phospholipid composition (Figure 3D). The results are consistent with the two-step mechanism of 1-acyl-G3P synthesis reported by Lu *et al*. [8], and show that the PlsX/Y pathway is essential for growth of *B. subtilis *lacking *plsB*.

### Functional relationship between *plsB, plsX *and *yneS/ygiH *in *E. coli*

It is generally believed that *E. coli plsB *is essential for bacterial growth, although experimental evidence supporting this hypothesis has yet to be obtained, and transposon mutagenesis experiments suggested that *plsX *is dispensable in *E. coli *[21]. The ortholog of *S. pneumoniae plsY *and *B. subtilis yneS *in *E. coli*, *ygiH*, is yet another dispensable gene [21]. Accordingly, we determined the functional relationship between *plsB*, *plsX*, and *ygiH*, in *E. coli*.

We initially attempted to systematically inactivate *E. coli *genes either singly or in combination. For this purpose, we replaced the *plsX *and *ygiH *genes with a kanamycin-resistance gene (*kan*) using the method of Datsenko and Wanner [22]. Inactivation of *plsB *was performed in the presence of plasmid-borne *plsB*. Phage P1 transduction frequencies of each allele into cells of various genetic backgrounds were measured to avoid possible effects of secondary mutations arising during gene inactivation (the *kan *cassette was removed, using FLP recombinase, in recipient cells for P1 transduction). The results, summarized in Table 1, show that *plsB *is essential for *E. coli *growth, while single deletions of either *plsX *or *ygiH *do not affect cell growth. These results suggested that PlsB plays a principal role in G3P acyltransferase activity in *E. coli*. Additionally, to examine whether *plsB *function might be complemented by overexpression of other genes, we introduced derivatives of plasmid pSTV28 or pSTV29 (12–15 copies per cell) harboring *E. coli plsX *and *ygiH*, and *B. subtilis plsX *and *yneS *into recipient strains for P1 transduction, without restoration of *plsB *function. Unexpectedly, although single deletions of *plsX *or *ygiH *did not affect cell growth, we found that the simultaneous inactivation of *plsX *and *ygiH *was impossible (Table 2), indicating that *plsX *and *ygiH *also have important roles in *E. coli *growth. Next, we expressed *B. subtilis plsX *and *yneS *genes in *E. coli*, and also the corresponding *E. coli *genes (as controls). The lethal phenotype of the *plsX *and *ygiH *double deletion was suppressed by *B. subtilis plsX *or *yneS*, indicating that these genes were able to complement the *plsX *and *ygiH E. coli *gene activities. In addition, a *plsX *and *ygiH *double deletion was possible when *plsB *was overexpressed from pSTV29*plsB*. To obtain further insights into the functional relationships between *plsB*, *plsX *and *ygiH*, we next introduced a plasmid harboring a mutated *plsB *gene (*plsB26*) into *E. coli*, and then attempted to inactivate *plsB*, *plsX *or *ygiH *(Table 3). The *plsB26 *gene has a point mutation that changes Gly1045 to Ala, reducing the affinity of PlsB26 for G3P [23]. In the presence of plasmid-borne *plsB26*, genomic *plsB *could be inactivated, indicating that PlsB26 protein expressed from the plasmid-borne gene could support *E. coli *growth. The plasmid-borne gene could not, however, complement the lethality of the *plsX *and *ygiH *double deletion. Furthermore, while native *plsB *could be inactivated in the presence of pSVT29*plsB26 *in cells lacking functional *ygiH*, *plsB *inactivation became impossible in cells with inactivated *plsX*, indicating that PlsX activity becomes indispensable for the growth of cells when the *plsB *activity is supplied by a plasmid copy of *plsB26*.

**Table 1:** Frequency of P1 transduction of *plsB*::*kan*, *plsX*::*kan*, and *ygiH*::*kan *mutations into W3110 cells harboring various plasmids

Recipient strain									Relative transduction frequency*		
	
	Chromosomal gene		Harboring plasmids						P1 donor strain		
			
Strain	*plsX*	*ygiH*	pSTV29	pSTV29 *plsB*	pSTV28 *EcplsX*	pSTV29 *ygiH*	pSTV28 *BsplsX*	pSTV29 *yneS*	Δ*plsB::kan*	Δ*plsX::kan*	Δ*ygiH::kan*
W3110	+	+	-	-	-	-	-	-	0	100	100
MEC104	+	+	+	-	-	-	-	-	0	50	64.3
MEC105	+	+	-	+	-	-	-	-	100	65.5	58.9
MEC106	+	+	-	-	+	-	-	-	0	80	50.9
MEC107	+	+	-	-	-	+	-	-	0	100	78
MEC108	+	+	-	-	-	-	+	-	0	63.6	100
MEC109	+	+	-	-	-	-	-	+	0	81.8	70.6

*Transduction frequencies are expressed as percentages of the highest number of Km^r ^colonies recorded in each experiment, and are depicted as blocks.

**Table 2:** Frequency of P1 transduction of *plsB*::*kan*, *plsX*::*kan*, and *ygiH*::*kan *mutations into strains MEC102 and MEC103 harboring various plasmids

Recipient strain									Relative transduction frequency*		
	
	Chromosomal gene		Harboring plasmids						P1 donor strain		
			
Strain	*plsX*	*ygiH*	pSTV29	pSTV29 *plsB*	pSTV28 *EcplsX*	pSTV29 *ygiH*	pSTV28 *BsplsX*	pSTV29 *yneS*	Δ*plsB::kan*	Δ*plsX::kan*	Δ*ygiH::kan*
MEC102	-	+	-	-	-	-	-	-	0	100	0
MEC103	+	-	-	-	-	-	-	-	0	0	100
MEC110	-	+	+	-	-	-	-	-	0	55.9	0
MEC111	-	+	-	+	-	-	-	-	100	53.5	59.8
MEC112	-	+	-	-	+	-	-	-	0	56.3	75.3
MEC113	-	+	-	-	-	+	-	-	0	83.1	100
MEC114	-	+	-	-	-	-	+	-	0	45.1	84.2
MEC115	-	+	-	-	-	-	-	+	0	100	92.7
MEC116	+	-	+	-	-	-	-	-	0	0	69.8
MEC117	+	-	-	+	-	-	-	-	100	63.7	85.9
MEC118	+	-	-	-	+	-	-	-	0	99.5	100
MEC119	+	-	-	-	-	+	-	-	0	85.1	73.8
MEC120	+	-	-	-	-	-	+	-	0	36.3	60.5
MEC121	+	-	-	-	-	-	-	+	0	100	96

*Transduction frequencies are expressed as percentages of the highest number of Km^r ^colonies recorded in each experiment, and are depicted as blocks.

**Table 3:** Frequency of P1 transduction of *plsB*::*kan*, *plsX*::*kan*, and *ygiH*::*kan *mutations into W3110, strain MEC102, and strain MEC103, harboring pSTV29 *-plsB *and *plsB26*

Recipient strain						Relative transduction frequency*		
	
	Chromosomal gene		Harboring plasmids			P1 donor strain		
			
Strain	*plsX*	*ygiH*	pSTV29	pSTV29 *plsB*	pSTV29 *plsB26*	Δ*plsB::kan*	Δ*plsX::kan*	Δ*ygiH::kan*
MEC104	+	+	+	-	-	0	66.4	76.9
MEC105	+	+	-	+	-	100	100	100
MEC288	+	+	-	-	+	61.9	67.7	55.1
MEC110	-	+	+	-	-	0	98.2	0
MEC111	-	+	-	+	-	100	75	100
MEC289	-	+	-	-	+	0	100	0
MEC116	+	-	+	-	-	0	0	100
MEC117	+	-	+	-	-	100	100	89
MEC290	+	-	-	-	+	59.3	0	61.7

*Transduction frequencies are expressed as percentages of the highest number of Km^r ^colonies recorded in each experiment, and are depicted as blocks.

**Figure 4:**
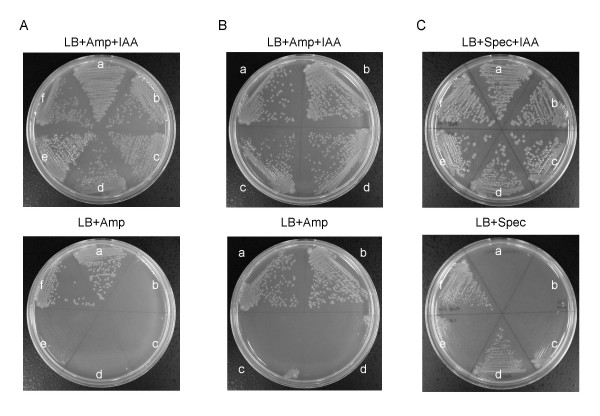
**Growth properties of *E. coli *strains harboring IAA-inducible genes**. *E. coli *strains harboring IAA-inducible genes were grown in LB medium containing 50 μg/ml ampicillin, with or without IAA (100 μg/ml), at 37°C. (A) a; MEC199 (Pw-*plsB*), b; MEC201 (Pw-*plsB *Δ*plsB*), c; MEC212 (Pw-*plsB *Δ*plsB *pSTV29 pMW118), d; MEC214 (Pw-*plsB *Δ*plsB *pSTV29 pMW118*yneS*), e; MEC218 (Pw-*plsB *Δ*plsB *pSTV29*BsplsX *pMW118), and f; MEC220 (Pw-*plsB *Δ*plsB *pSTV29*BsplsX *pMW118*yneS*). (B) a; MEC308 (Pw-*EcplsX*), b; MEC309 (Pw-*ygiH*), c; MEC306 (Pw-*EcplsX ΔplsX ΔygiH*), and d; MEC307 (Pw-*ygiH ΔplsX ΔygiH*). (C) *E. coli *strains harboring pUC18S or pUC18S *tesA *were grown in LB medium containing 50 μg/ml spectinomycin, with or without IAA (50 μg/ml), at 30°C. a; MEC323 (Pw-*plsB *Δ*plsB *Δ*ygiH *pUC18S), b; MEC324 (Pw-*plsB *Δ*plsB *Δ*ygiH *pUC18S *tesA*), c; MEC325 (Pw-*EcplsX ΔplsX ΔygiH *pUC18S), d; MEC326 (Pw-*EcplsX ΔplsX ΔygiH *pUC18S *tesA*), e; MEC327 (Pw-*ygiH ΔplsX ΔygiH *pUC18S), e; MEC328 (Pw-*ygiH ΔplsX ΔygiH *pUC18S *tesA*).

The essential nature of *plsB*, and the lethality of the *plsX *and *ygiH *double deletion, were further confirmed using inducible forms of these genes. To this end, we placed the coding sequences of *plsB, plsX *and *ygiH *under the control of an IAA (3β-indoleacrylic acid)-inducible *trp *promoter (Pw), integrated the genes into the chromosome as described in Methods, and then deleted the native copies of either *plsB *alone, or both *plsX *and *ygiH*. As expected, strains MEC201 (Pw-*plsB, ΔplsB*), MEC306 (Pw-*plsX, ΔplsX, ΔygiH*) and MEC307 (Pw-*ygiH, ΔplsX*, Δ*ygiH*) cells displayed IAA-dependent growth (Figure 4A and 4B).

The PlsB and PlsX enzymes utilize acyl-ACP as acyl-donor, and abnormal accumulation of acyl-ACP has been shown to result in growth impairment [24]. To examine acyl-ACP accumulation in cells with inactivated *plsX *and *ygiH *genes, we introduced a derivative of pUC18, harboring the *E. coli tesA *gene encoding thioesterase I [25], into cells of strains MEC306 and MEC307. Interestingly, the growth defects of these strains in the absence of IAA were suppressed by the additional supply of thioesterase I (TesA hydrolyses acyl-ACP) (Figure 4C). We have not yet succeeded in demonstrating the expected TesA-dependent suppression of the inability to achieve *plsX *inactivation when *plsB *activity is supplied by a plasmid encoding *plsB26*. This is because it is difficult to construct an appropriate strain in which to examine the hypothesis.

### Complementation of the PlsB activity by a combination of PlsX and YneS/YgiH

As we found that the combination of PlsX and YneS conferred the ability to synthesize 1-acyl-G3P upon *B. subtilis*, we expected that simultaneous overexpression of these two proteins would complement a *plsB *mutation in *E. coli*. To test this hypothesis, we introduced various combinations of plasmids harboring *plsX *and *yneS*/*ygiH *into *E. coli *cells. In these experiments, we employed derivatives of plasmid pMW118 (ca. 5 copies per cell), whose replication is compatible with that of pSTV28/29 derivatives, when more than one cloned gene was to be expressed. We successfully complemented the *E. coli plsB *mutation by expression of *B. subtilis plsX *and *yneS *(Table 4). The complementation of the *E. coli *PlsB activity by the *B. subtilis *PlsX and YneS combination was also demonstrated using an IAA-inducible *plsB *gene (Figure 4A). Other gene combinations, including those with *E. coli *genes, did not complement *plsB*. Plasmid pMW118*ygiH *complemented the growth defect of MEC306 and MEC307 cells in the absence of IAA (data not shown), indicating that functional YgiH is expressed from pMW118*ygiH*. Although we could not determine the expression levels of the various proteins due to the lack of appropriate antibodies, the specific activities of *E. coli *PlsX and YgiH may be lower than those of their *B. subtilis *counterparts.

**Table 4:** Frequency of P1 transduction of *plsB*::*kan*, *plsX*::*kan*, and *ygiH*::*kan *mutations into W3110 cells harboring various pairs of plasmids.

Recipient strain									Relative transduction frequency*		
	
	Chromosomal gene		Harboring plasmids						P1 donor strain		
			
Strain	*plsX*	*ygiH*	pSTV29	pSTV28 *EcplsX*	pSTV28 *BsplsX*	pMW118	pMW118 *ygiH*	pMW118 *yneS*	Δ*plsB::kan*	Δ*plsX::kan*	Δ*ygiH::kan*
MEC130	+	+	+	-	-	+	-	-	0	56.1	39.4
MEC134	+	+	+	-	-	-	+	-	0	53.7	41.3
MEC136	+	+	+	-	-	-	-	+	0	100	81.7
MEC137	+	+	-	+	-	+	-	-	0	87.8	69.2
MEC139	+	+	-	+	-	-	+	-	0	73.2	100
MEC140	+	+	-	-	+	+	-	-	0	90.2	46.2
MEC142	+	+	-	-	+	-	-	+	100	97.6	90.4

*Transduction frequencies are expressed as percentages of the highest number of Km^r ^colonies recorded in each experiment, and are depicted as blocks.

## Discussion

Our genetic studies have shown that PlsX and YneS (now renamed PlsY) are essential for G3P acyltransferase activity in *B. subtilis *cells, and that the combination of the two *B. subtilis *proteins complements PlsB activity in *E. coli *cells. Our findings are consistent with the two-step mechanism of 1-acyl-G3P synthesis reported by Lu *et al*. [8], and we have shown that the PlsX/Y pathway is essential for the growth of *B. subtilis *lacking *plsB*. The combination of *plsX *and *plsY *is widely conserved in eubacteria, but most eubacteria lack *plsB *[8], strongly suggesting that *plsX/Y *will be essential genes in these bacteria.

A number of bacteria, belonging mainly to the gamma group of proteobacteria, possess both *plsB *and *plsX*/*plsY*, however. Another important finding reported here is that, in such cases, the relationship between the two 1-acyl-G3P synthesis pathways may be variable and complex. Although *plsB *has been reported to be dispensable in *Pseudomonas aeruginosa *[26], we found that the gene is indispensable in *E. coli*, indicating that PlsB is principally responsible for 1-acyl-G3P synthesis in *E. coli*. On the other hand, we found that simultaneous inactivation of both PlsX and PlsY is impossible, and that this lethality was complemented by overexpression of PlsB. As single deletions in either *plsX *or *ygiH *did not affect cell growth, the lethality of the *plsX *and *ygiH *double deletion cannot be because of inactivation of a minor pathway (using acyl-PO_4_) for 1-acyl-G3P synthesis.

Interestingly, PlsX activity becomes indispensable for growth of *E. coli *cells in which PlsB activity is supplied from a plasmid copy of *plsB26*. Growth, in minimal medium, of *E. coli *SJ22 cells harboring the *plsB *mutation on the chromosome is dependent on a supply of exogenous G3P. Depletion of G3P in the growth medium has been found to result in accumulation of acyl-ACP due to the blockage of 1-acyl-G3P synthesis, leading to a severe reduction in fatty acid biosynthesis because accumulated acyl-ACP causes feedback inhibition of enzymes in the biosynthetic pathway [27,28]. G3P-acyltransferase activity expressed from a plasmid-borne copy of *plsB26 *may be less than that in wild type cells, and acyl-ACP may therefore accumulate in such cells. PlsX may be able to suppress the deleterious accumulation of acyl-ACP by converting acyl-ACP to acyl-PO_4_. PlsY may also be involved in control of the intracellular acyl-ACP concentration. *E. coli *PlsB has been shown to utilize both acyl-ACP and acyl-CoA as acyl donors in the synthesis of 1-acyl-G3P [7], but does not use these donors to make acyl-PO_4 _[8]. On the other hand, *S. pneumoniae *PlsY has been shown to synthesize 1-acyl-G3P using both acyl-ACP and acyl-PO_4_. These results suggest that *E. coli *PlsY, in the absence of PlsX, may synthesize 1-acyl-G3P using acyl-ACP, and this activity would then contribute to consumption of acyl-ACP. It is therefore possible that both PlsX and PlsY contribute independently to the maintenance of an appropriate intracellular acyl-ACP concentration, and simultaneous inactivation of both proteins therefore results in deleterious accumulation of acyl-ACP. Overexpression of PlsB increases 1-acyl-G3P synthesis activity and may suppress the accumulation of acyl-ACP resulting from the *plsX *and *ygiH *double deletion. Suppression of growth defects due to the *plsX *and *ygiH *double deletion by overexpression of thioesterase I strongly supports our hypothesis.

Acyl-ACP has been shown to be not only a central cofactor for fatty acid and phospholipid synthesis, but also a regulatory molecule, coordinating the synthesis of these lipids with cell growth. Our results strongly suggest that PlsX, PlsY and PlsB form a complex network functioning to supply appropriate levels of lipid biosynthetic precursors, especially acyl-ACP. In turn, this controls the synthesis of 1-acyl-G3P in *E. coli *cells, resulting in appropriate levels of 1-acyl-G3P under various growth conditions. Further studies are needed to reveal the precise roles of PlsX, PlsY and PlsB.

## Conclusion

Both *plsX *and *yneS *are essential for 1-acyl-G3P synthesis in *B. subtilis *cells lacking *plsB*, in agreement with recent reports on the biochemical functions of these genes. Both genes will be essential for bacterial growth lacking *plsB*. In *E. coli*, PlsB plays a principal role in 1-acyl-G3P synthesis and is also essential for bacterial growth. PlsX and YgiH also, however, play important roles in *E. coli *growth, probably by regulating the intracellular concentrations of acyl-ACP at appropriate levels. These proteins are therefore important targets for the development of new antibacterial reagents.

## Methods

### Materials and strains

The *B. subtilis *and *E. coli *strains used in this study are listed in Tables S1 [see Additional file 1] and S2 [see Additional file 2], respectively. Primers and plasmids are specified in Tables S3 [see Additional file 3] and S4 [see Additional file 4], respectively. *B. subtilis *cells were transformed as described previously [29]. *E. coli *strains DH5α (Takara) and JM105 (Takara), were used throughout as cloning hosts.

Full-length sequences of *E. coli plsB*, *plsX *(*EcplsX*), and *ygiH*, and *B. subtilis plsX *(*BsplsX*) and *yneS*, were amplified and cloned into plasmids pSTV28 or pSTV29 (Takara, 12–15 copies per cell) as summarized in Table S3 [see Additional file 3]. A DNA fragment containing a mutated *plsB *sequence, *plsB26*, was PCR-amplified from genomic DNA of *E. coli *strain TL84 [3], and cloned into pSTV29. Inserts of pSTV29*ygiH *and pSTV29*yneS *were transferred into pMW118 (Nippon Gene, ca. 5 copies per cell), because the replication of pMW118 is compatible with that of pSTV28 and pSTV29.

The IAA (3β-indoleacrylic acid, Sigma)-inducible *trp *promoter fragment was PCR amplified from W3110 chromosomal DNA. The PCR product was digested with the restriction enzymes *Mun*I and *Eco*RI, and cloned into the *Eco*RI site of pMC1403 [30], generating pMC1403Pw. Full-length sequences of *E. coli plsB*, *plsX*, and *ygiH *were amplified and cloned into the *Eco*RI/*Bam*HI, *Bam*HI, or *Eco*RI/*Bam*HI sites of pMC1403-Pw, respectively, to obtain pMC1403Pw-*plsB*, pMC1403Pw-*plsX *and pMC1403Pw-*ygiH*.

The full-length sequence of *E. coli tesA *was amplified and cloned into plasmid pUC18S. Plasmid pUC18S was created by replacing the *Aat*II/*Eam*1105I fragment (containing the ampicillin-resistance gene) of pUC18 (Takara) with an *Aat*II/*Eam*1105I fragment (containing the spectinomycin-resistance gene) amplified, by PCR, from pAPNC213 [17].

*B. subtilis *strains were grown in Luria-Bertani (LB) medium, or DSM, supplemented with chloramphenicol (5 μg/ml), kanamycin (5 μg/ml), or erythromycin (0.5 μg/ml), as appropriate.*E. coli *strains were grown in LB or M9 minimal media, supplemented with ampicillin (50 μg/ml), chloramphenicol (5 μg/ml), spectinomycin (50 μg/ml), or kanamycin (25 μg/ml), as appropriate.

### Isolation of the *B. subtilis yneS-ts *mutant

The *yneS-ts *mutant was isolated according to the procedure of F. Kawamura (personal communication). The *B. subtilis *strain MY101, in which the sequence 45–103 bp downstream of the termination codon of *yneS *(in the coding sequence of *yneR*, which is downstream of *yneS*) was replaced with a *cat *gene, was generated as follows. The regions upstream and downstream of the insertion site were amplified by PCR using a yneS-F1 and yneS-R1 primer pair, and a yneS-F2 and yneS-R2 primer pair, respectively. The *cat *gene of pCBB31 [31] was amplified using primers cat-F and cat-R. The primer pairs yneS-R1 and cat-R, and cat-F and yneS-F2, contain overlapping sequences, and the three fragments obtained were ligated by recombinant PCR using yneS-F1 and yneS-R2 primers. *B. subtilis *strain 168 was transformed with the resulting fragment, and chloramphenicol-resistant transformants were selected (MY101, Δ*yneR*::*cat*^+^). Next, the pCH11 plasmid containing an inactive *cat *gene (a nonsense mutation is located in the gene), a kanamycin-resistance gene, and a *ts *origin of replication, was introduced into MY101. We selected for kanamycin resistance and chloramphenicol sensitivity in order to isolate cells with the inactive *cat *gene on the chromosome, which occurred when gene conversion from the plasmid copy was successful [32]. The introduced plasmid was removed by cultivation at the temperature restrictive for pCH11 replication, to generate strain MY102 (Δ*yneR*::*cat*^-^). PCR mutagenesis of the *yneS-cat *fragment was performed using r*Taq *polymerase (Takara) with MY101 chromosomal DNA as template. The mutated *yneS-cat *fragment was used to transform strain MY102. Chloramphenicol-resistant transformants were selected at 30°C, and colonies that were unable to grow above 42°C were screened to isolate strain MY103 (*yneS-ts*).

### Construction of the *B. subtilis plsC-ts *mutant

As the growth of cells in which *gfp *(encoding green fluorescent protein) was translationally fused to the C-terminus of PlsC became temperature-sensitive (data not shown), we predicted that the required *plsC*-*ts *mutant could be obtained by modification of the C-terminal region of the *plsC *gene. We therefore created a series of mutants with deletions of different numbers of amino acids from the C-terminus of PlsC and found that deletion of the 7 end residues resulted in a *ts *phenotype (strain MY112). To generate strain MY112, the 3' end of the *plsC *coding region (with the 7 codons deleted) was amplified, and a termination codon was added by PCR using the primers plsC-F1 and plsC-CΔ7-R1. The region downstream of *plsC *was amplified with the oligonucleotides plsC-F2 and plsC-R2. The two fragments were ligated to the 5' and 3' ends of the *cat *gene (amplified from pCBB31 using plsC-cat-F and plsC-cat-R primers) by recombinant PCR with overlapping sequences contained within the plsC-CΔ7-R1 and plsC-cat-F primer pair, and the plsC-F2 and plsC-cat-R primer pair. The resulting fragment was used to transform *B. subtilis *strain 168 with selection for chloramphenicol resistance.

### Construction of an IPTG-dependent mutant of *B. subtilis plsX*

The *plsX *gene is located in the fatty acid biosynthesis operon composed of *fapR*, *plsX*, *fabD*, *fabG*, and *acpA*. To generate cells in which *plsX *expression was under the control of the IPTG-inducible Pspac promoter, we initially integrated Pspac-*plsX *into the *aprE *locus of the bacterial chromosome. The *plsX *gene was amplified by PCR using the pAP-plsX-F and pAP-plsX-R primers, and inserted between the Pspac promoter and the kanamycin-resistance gene (*kan*) on pAPNCK, that contains sequences upstream and downstream of *aprE*, flanking Pspac and *kan*. Plasmid pAPNCK was created by replacing the *Hind*III fragment (containing the spectinomycin-resistance gene) of pAPNC213 [17] with the *Hind*III fragment (containing the *kan *gene) of pDG780 [33]. The resulting plasmid was transformed into *B. subtilis *cells and selection of a double crossover using kanamycin resistance yielded strain MY110 (Δ*aprE*::Pspac-*plsX-kan*). Next, the native *plsX *gene was replaced with the *cat *gene, without the promoter and terminator sequences, to ensure the correct expression of downstream essential genes. To achieve this, upstream and downstream regions of *plsX *were amplified using the plsX-F1and plsX-R1 primer pair, and the plsX-F2 and plsX-R2 primer pair, respectively. Fragments were ligated to the 5' and 3' ends of the *cat *gene (amplified from pCBB31 using primers cat-F-p and cat-R-t) by recombinant PCR using overlapping sequences in the plsX-R1 and cat-F-p primer pair, and the cat-R-t and plsX-F2 primer pair. The resulting fragment was used to transform strain MY110, and chloramphenicol-resistant transformants were selected to generate strain MY111 (Δ*aprE*:: Pspac-*plsX-kan *Δ*plsX:*:*cat*-p-t).

### Analysis of phospholipid composition

Cells of *B. subtilis *strains 168, *yneS-ts *(MY103), and *plsC*-*ts *(MY112) were cultured in LB medium at 30°C until an OD_600 _of 0.4 was attained, and the temperature was then shifted to 42°C. Cells were pulse-labeled with ^32 ^[Pi] (50 μCi/ml, Amersham Biosciences) for 5 min after 1 h of cultivation at 42°C. Incorporation of the label was terminated by the addition of 0.8 ml of culture to 3.0 ml of chloroform:methanol (1:2, v/v). Lipids were extracted using the method of Bligh and Dyer [20]. The phospholipids produced were examined by two-dimensional thin-layer chromatography on Silica Gel 60 plates (Merck) developed with chloroform:methanol:water (65:25:4) in the first dimension and chloroform:methanol:acetic acid (65:25:10) in the second dimension [5]. The incorporation of label into lysophosphatidic acid and other lipids was determined and quantitated using the BAS2500 image analyzer (Fuji).

Pspac-*plsX *(MY111) cells were cultured in LB medium containing 50 μM IPTG at 37°C until an OD_600 _value of 0.4 was attained. Cells were harvested, washed twice with LB medium, and suspended in 5 mL LB medium with or without 1 mM IPTG at final densities (OD_600 _values) of 0.06 or 0.1, respectively. Next, cells were pulse-labeled with ^32 ^[Pi] (50 μCi/ml) for 5 min after 1 h of cultivation at 37°C. Lipid extraction and analysis were performed as described above.

### Gene disruption in *E. coli*

Deletions of the *plsB*, *plsX*, and *ygiH *genes were achieved using a one-step chromosomal gene inactivation method involving the phage λ Red recombination system, as developed by Datsenko and Wanner [22,34]. The *E. coli *strain, BW25113, was used. The primers used for target gene disruptions are listed in Table S4 [see Additional file 4]. The 5' end of each primer contained 60–70 bp of sequences upstream or downstream of the target gene, while the 3' end contained specific 20-nucleotide sequences from the template, pKD13. The primer pairs plsBdelup and plsBdeldown, plsXdelup and plsXdeldown, and ygiHdelup and ygiHdeldown, were used to generate Δ*plsB*::*kan *(in strain MEC001), Δ*plsX*::*kan *(in strain MEC002), and Δ*ygiH*::*kan *(in strain MEC003) mutations, respectively. Because *E. coli plsB *was essential for growth, inactivation of *plsB *was performed in the presence of a plasmid harboring the gene (pSTV28*plsB-p*). Deletion mutations were confirmed using colony PCR with a *kan*-specific primer (k1 or k2) [22] and the locus-specific primers listed in Table S4 [see Additional file 4]. The deletion mutations, Δ*plsX*::*kan *and Δ*ygiH*::*kan*, were transferred into prototrophic strain W3110 by P1*vir *transduction to obtain strains MEC005 and MEC006, respectively. The *kan *cassettes in strains MEC005 and MEC006 were removed with FLP recombinase, expressed by plasmid pCP20, as described previously [22], to obtain strains MEC102 and MEC103, respectively.

### Construction of *E. coli *strains harboring IAA-inducible genes

To introduce *plsB*,*plsX *and *ygiH *genes, under the control of IAA-inducible *trp *promoters, into the *E. coli *chromosome, cloned gene fragments in pMC1403-based plasmids were transferred into the λRZ5 phage by *recA*-mediated homologous recombination as previously described [35]. The technique involves double crossovers between common upstream ampicillin-resistance genes and downstream *lac *operon sequences. The resulting λPw-*plsB*, λPw-*plsX*, and λPw-*ygiH *phages were lysogenized into the W3110 chromosome, to obtain strains MEC199, MEC308, and MEC309, respectively. The Δ*plsB*::*kan *mutation was transferred into strain MEC199 by P1*vir *transduction, and the *kan *cassette was then removed with FLP recombinase, expressed from plasmid pCP20, to obtain strain MEC201. The λPw-*plsX *and λPw-*ygiH *phages were also lysogenized into the strain MEC102 chromosome, to generate strains MEC303 and MEC304, respectively. The Δ*ygiH*::*kan *mutation was then transferred into strains MEC303 and MEC304 by P1*vir *transduction.

## Abbreviations

G3P, Glycerol-3-phosphate; 1-acyl-G3P, 1-acyl-glycerol-phosphate; PA, phosphatidic acid; PG, phosphatidylglycerol; PE, phosphatidylethanolamine; CL, cardiolipin; IAA, 3β-indoleacrylic acid.

## Authors' contributions

All experimental work was carried out by MY under the supervision of TO and NO. TO provided the *E. coli *transduction system. MY and TO wrote the draft manuscript. NO finalized the analysis and completed the manuscript. All authors read and approved the final manuscript.

## Supplementary Material

Additional file 1 *B. subtilis *strains used in this study, and their genotypes. List of *B. subtilis *strains used in this study. Click here for file

Additional file 2 *E. coli *strains used in this study, and their genotypes. List of *E. coli *strains used in this study. Click here for file

Additional file 3 Plasmids used in this study. List of plasmids used in this study. Click here for file

Additional file 4 Oligonucleotides used in this study. List of oligonucleotides used in this study. Click here for file
