# Supplementary material for: Involvement of the YneS/YgiH and PlsX proteins in phospholipid biosynthesis in both Bacillus subtilis and Escherichia coli
Source: BMC Microbiol. 2007 Jul 24;7:69. doi: 10.1186/1471-2180-7-69 (PMC1950310; doi:10.1186/1471-2180-7-69)
Supplement: Additional file 1 — B. subtilis strains used in this study, and their genotypes. List of B. subtilis strains used in this study. [file 1471-2180-7-69-S1.pdf]

Table S1 *B. subtilis* strains used in this study and their genotypes

Strain	Genotype	Construction	Reference or source
168	*trpC2*		Laboratory stock
YNESp	*trpC2* Δ*yneS*::pMutinNC (Pspac-*yneS erm*)		(1)
MY101	*trpC2 yneR*::*cat*$^{+}$		This study
MY102	*trpC2* Δ*yneR*::*cat*$^{-}$	pCH11 –> MY101	This study
MY103	*trpC2 yneS-ts cat*		This study
MY105	*trpC2 yneS-ts cat sup-1*		This study
MY107	*trpC2* Δ*yneS*::pMutinNC (*Pspac-yneS erm*) *sup-1*	YNESp –> MY105	This study
MY108	*trpC2* Δ*glpD*::pMutinNC (*erm*)	pMutinNC*glpD* –> 168	This study
MY109	*trpC2 yneS-ts cat* Δ*glpD*::pMutinNC *(erm*)	MY108 –> MY103	This study
MY110	*trpC2* Δ*aprE*::Pspac-*plsX-kan*	pAPNCKplsX –> 168	This study
MY111	*trpC2* Δ*aprE*::Pspac-*plsX-kan* Δ*plsX*::*cat-p-t**		This study
MY112	*trpC2 plsC*-CΔ7 *cat*		This study

* coding sequence of the chloramphenicol-resistant gene without the promoter and terminator

Reference

1. Kobayashi K, Ehrlich SD, Albertini A, Amati G, Andersen KK, Arnaud M, Asai K, Ashikaga S, Aymerich S, Bessieres P *et al*: **Essential *Bacillus subtilis* genes**. *Proc Natl Acad Sci U S A* 2003, **100**(8):4678-4683.
