# Supplementary material for: Involvement of the YneS/YgiH and PlsX proteins in phospholipid biosynthesis in both Bacillus subtilis and Escherichia coli
Source: BMC Microbiol. 2007 Jul 24;7:69. doi: 10.1186/1471-2180-7-69 (PMC1950310; doi:10.1186/1471-2180-7-69)
Supplement: Additional file 2 — E. coli strains used in this study, and their genotypes. List of E. coli strains used in this study. [file 1471-2180-7-69-S2.pdf]

Table S2 *E. coli* strains used in this study and their genotypes

Strain	Genotype	Construction	Reference or source
DH5a	*supE44* Δ*lacU169 (f80lacZ*Δ*M 15)hsdR17 recA1 endA1 gyrA96 thi-1 relA1*		Laboratory stock
JM105	*endA1 supE sbcB15 thi rpsL* Δ*(lac-proAB)/F' [traD36* $proAB^{+}$ $lacI^{q}$ *lacZ* Δ*M15]*		Laboratory stock
W3110			Laboratory stock
BW25113	$lacI^{q}$ $rrnB_{T14}$ $\Delta lacZ_{WJ16}$ *hsdR514* $\Delta araBAD_{AH33}$ $\Delta rhaBAD_{LD78}$		Laboratory stock
TL84	HfrC *phoA8 glpD3 glpR2 relA1 spoT1 pit-10 fhuA22 ompF627 fadL701* (l) $glpK^{i}$ *plsB26 plsX50 zjb-750*::Tn*10*		(1)
MEC001	BW25113 pSTV29-*plsB-p* Δ*plsB*::*kan*		This study
MEC002	BW25113 Δ*plsX*::*kan*		This study
MEC003	BW25113 Δ*ygiH*::*kan*		This study
MEC005	W3110 Δ*plsX*::*kan*	P1 MEC002 –> W3110	This study
MEC006	W3110 Δ*ygiH*::*kan*	P1 MEC003 –> W3110	This study
MEC102	W3110 Δ*plsX*	pCP20 –> MEC002	This study
MEC103	W3110 Δ*ygiH*	pCP20 –> MEC003	This study
MEC104	W3110 pSTV29		This study
MEC105	W3110 pSTV29*plsB*		This study
MEC106	W3110 pSTV28*EcplsX*		This study
MEC107	W3110 pSTV29*ygiH*		This study
MEC108	W3110 pSTV28*BsplsX*		This study
MEC109	W3110 pSTV29*yneS*		This study
MEC110	MEC102 pSTV29		This study
MEC111	MEC102 pSTV29*plsB*		This study
MEC112	MEC102 pSTV28*EcplsX*		This study
MEC113	MEC102 pSTV29*ygiH*		This study
MEC114	MEC102 pSTV28*BsplsX*		This study
MEC115	MEC102 pSTV29*yneS*		This study
MEC116	MEC103 pSTV29		This study
MEC117	MEC103 pSTV29*plsB*		This study
MEC118	MEC103 pSTV28*EcplsX*		This study
MEC119	MEC103 pSTV29*ygiH*		This study
MEC120	MEC103 pSTV28*BsplsX*		This study
MEC121	MEC103 pSTV29*yneS*		This study
MEC130	W3110 pSTV29 + pMW118		This study
MEC134	W3110 pSTV29 + pMW118*ygiH*		This study
MEC136	W3110 pSTV29 + pMW118*yneS*		This study
MEC137	W3110 pSTV28*EcplsX* + pMW118		This study
MEC139	W3110 pSTV28*EcplsX* + pMW118*ygiH*		This study
MEC140	W3110 pSTV28*BsplsX* + pMW118		This study
MEC142	W3110 pSTV28*BsplsX* + pMW118*yneS*		This study
MEC199	W3110 λpMC1403Pw-*plsB*		This study
MEC200	MEC199 Δ*plsB*::*kan*	P1 MEC001 –> MEC199	This study

Table S2 *E. coli* strains used in this study and their genotypes (continued)

Strain	Genotype	Construction	Reference or source
MEC201	MEC200 Δ*plsB*	pCP20 –> MEC200	This study
MEC203	MEC103 λpMC1403 Pw-*plsB*		This study
MEC205	MEC203 Δ*plsB*::*kan*	P1 MEC003 -> MEC203	This study
MEC212	MEC201 pSTV29 + pMW118		This study
MEC214	MEC201 pSTV29 + pMW118*yneS*		This study
MEC218	MEC201 pSTV28*BsplsX* + pMW118		This study
MEC220	MEC201 pSTV28*BsplsX* + pMW118*yneS*		This study
MEC288	W3110 pSTV29*plsB26*		This study
MEC289	MEC102 pSTV29*plsB26*		This study
MEC290	MEC103 pSTV29*plsB26*		This study
MEC303	MEC102 λpMC1403Pw-*EcplsX*		This study
MEC304	MEC102 λpMC1403Pw-*ygiH*		This study
MEC306	MEC306 Δ*ygiH*::*kan*	P1 MEC003 –> MEC306	This study
MEC307	MEC307 Δ*ygiH*::*kan*	P1 MEC003 –> MEC307	This study
MEC308	W3110 λpMC1403Pw-*EcplsX*		This study
MEC309	W3110 λpMC1403Pw-*ygiH*		This study
MEC323	MEC205 pUC18S		This study
MEC324	MEC205 pUC18S*tesA*		This study
MEC325	MEC306 pUC18S		This study
MEC326	MEC306 pUC18S*tesA*		This study
MEC327	MEC307 pUC18S		This study
MEC328	MEC307 pUC18S*tesA*		This study

Reference

1. Larson TJ, Ludtke DN, Bell RM: **sn-Glycerol-3-phosphate auxotrophy of *plsB* strains of *Escherichia coli*: evidence that a second mutation, *plsX*, is required**. *J Bacteriol* 1984, **160**(2):711-717.
