# Supplementary material for: Involvement of the YneS/YgiH and PlsX proteins in phospholipid biosynthesis in both Bacillus subtilis and Escherichia coli
Source: BMC Microbiol. 2007 Jul 24;7:69. doi: 10.1186/1471-2180-7-69 (PMC1950310; doi:10.1186/1471-2180-7-69)
Supplement: Additional file 3 — Plasmids used in this study. List of plasmids used in this study. [file 1471-2180-7-69-S3.pdf]

Table S3 Plasmids used in this study

Plsamid	Characteristic	Primers for construction	Reference or Source
**Plasmids used for the *B. subtilis* transformation**			
pCH11	pUB110*ori-ts* pBR322*ori cat*$^-$(harboring nonsense mutation) (Km^r Amp^r)		(1)
pCA191	pUC19 derivative harboring *cat* (Cm^r Amp^r)		(2)
pCA191*glpD*-FL	pCA191 derivative harboring full length of *glpD* (-288 to 1668) (Cm^r)	glpD-N-F, glpD-C-R	This study
pCA191*glpD*-N	pCA191 derivative harboring N-terminal fragment of *glpD* (-288 to 942) (Cm^r)	glpD-N-F, glpD-N-R	This study
pCA191*glpD*-C	pCA191 derivative harboring C-terminal fragment of *glpD* (820 to 1668) (Cm^r)	glpD-C-F, glpD-C-R	This study
pMutinNC	Plasmid vector to construct knockout or conditional-null mutants of the *B. subtilis* genes (Em^r Amp^r)		(3)
pMutinNC*glpD*	pMutNC derivative harboring internal fragment of *glpD* (23 to 182) (Em^r)	pMut-glpD-F, pMut-glpD-R	This study
pAPNC213	Plasmid vector to integrate a target gene fused with P*spac* into the *B. subtilis aprE* locus (Spec^r Amp^r)		(3)
pDG780	pBluescript KS$^+$ harboring *kan* (Amp^r Km^r)		(4)
pAPNCK	pANNC213 derivative harboring *kan* (Km^r Amp^r)		This study
pAPNCK*plsX*	pAPNCK derivative harboring *plsX* (-22 to 1011) (Km^r)	pAP-plsX-F, pAP-plsX-R	This study
**Plasmids used for protein expression in *E. coli***			
pSTV28	pACYC184 derived cloning vector (Cm^r)		Takara
pSTV29	pACYC184 derived cloning vector (Cm^r)		Takara
pSTV29*plsB*	pSTV29 derivative harboring *plsB* (-229 to 2428) (Cm^r)	PLSB-F, PLSB-R	This study
pSTV28*EcplsX*	pSTV29 derivative harboring *E.coli plsX* (-81 to 1045) (Cm^r)	ECPLSX-F, ECPLSX-R	This study
pSTV29*ygiH*	pSTV29 derivative harboring *ygiH* (-177 to 625) (Cm^r)	YGIH-F, YGIH-R	This study
pSTV28*BsplsX*	pSTV29 derivative harboring *B.subtilis plsX* (-22 to 1011) (Cm^r)	BSPLSX-F, BSPLSX-R	This study
pSTV29*yneS*	pSTV29 derivative harboring *yneS* (-129 to 584) (Cm^r)	YNES-F, YNES-R	This study
pSTV28*plsB-p*	pSTV28 derivative harboring *plsB* (-90 to 2424) (Cm^r)	PLSB-p-F, PLSB-p-R	This study
pMW118	pSC101 derived cloning vector (Amp^r)		Nippon Gene
pMW118*ygiH*	pMW118 derivative harboring *ygiH* (-177 to 625) (Amp^r)		This study
pMW118*yneS*	pMW118 derivative harboring *yneS* (-129 to 584) (Amp^r)		This study
pSTV29*plsB26*	pSTV29 derivative harboring *plsB26* (-169 to 2488) (Cm^r)	PLSB-F, PLSB-R	This study
pUC18	pMB1 derived cloning vector (Amp^r)		Takara
pUC18S	pUC18 derivative harboring *spec* (Spec^r)	Spec-F-AatII, Spec-R-Eam1105I	This study
pUC18S*tesA*	pUC18S derivative harboring *tesA* (-82 to 638) (Spec^r)	TesA-FEcoRI, TesA-RBamHI	This study
**Plasmids used for construction of IAA-inducible strain in *E. coli***			
pMC1403	pBR322 derived *lacZ* fusion vector (Amp^r)		(5)
pMC1403Pw	pMC1403 derivative harboring the *trp* promoter region (*trpL* -105 to 120) (Amp^r)	Ptrp-FMunI, Ptrp-REcoRI	This study
pMC1403Pw-*plsB*	pMC1403Pw derivative harboring *plsB* (-20 to 2424) (Amp^r)	PLSB-F2, PLSB-R2	This study
pMC1403Pw-*EcplsX*	pMC1403Pw derivative harboring *E.coli plsX* (-81 to 1045) (Amp^r)	ECPLSX-F2, EC-PLSX-R2	This study
pMC1403Pw-*ygiH*	pMC1403Pw derivative harboring *ygiH* (-16 to 625) (Amp^r)	YGIH-F2, YGIH-R2	This study

References

1. Kawamura, F., personal communication

2. Murakami T, Haga K, Takeuchi M, Sato T: **Analysis of the *Bacillus subtilis spoIIIJ* gene and its Paralogue gene, *yqjG***. *J Bacteriol* 2002, **184**(7):1998-2004.

3. Morimoto T, Loh PC, Hirai T, Asai K, Kobayashi K, Moriya S, Ogasawara N: **Six GTP-binding proteins of the Era/Obg family are essential for cell growth in *Bacillus subtilis***. *Microbiology* 2002, **148**(Pt 11):3539-3552.

4. Guerout-Fleury AM, Shazand K, Frandsen N, Stragier P: **Antibiotic-resistance cassettes for *Bacillus subtilis*.** Gene 1995, **167**(1-2):335-336.

5. Casadaban MJ, Chou J, Cohen SN: **In vitro gene fusions that join an enzymatically active ß-galactosidase segment to amino-terminal fragments of exogenous proteins: *Escherichia coli* plasmid vectors for the detection and cloning of translational initiation signals**. *J Bacteriol* 1980, **143**(2):971-980.
