# Supplementary material for: Involvement of the YneS/YgiH and PlsX proteins in phospholipid biosynthesis in both Bacillus subtilis and Escherichia coli
Source: BMC Microbiol. 2007 Jul 24;7:69. doi: 10.1186/1471-2180-7-69 (PMC1950310; doi:10.1186/1471-2180-7-69)
Supplement: Additional file 4 — Oligonucleotides used in this study. List of oligonucleotides used in this study. [file 1471-2180-7-69-S4.pdf]

Table S4 Oligonucleotides used in this study

Oligonucleotides	Sequence (5' to 3')
**Construction of *B. subtilis* mutants**	
yneS-F1	TACTTGATAGGCAGCATTCC
yneS-R1	CGTCGTGACTGGGAAAACGTATTAGGCTATTCTGTGTC
yneS-F2	GTTATCCGCTCACAATTCACGAAGACGCATTGAACTGG
yneS-R2	GAAGTGAACGGGATATGGCG
cat-F	GAATTGTGAGCGGATAAC
cat-R	GTTTTCCCAGTCACGACG
glpD-N-F	GCGAATTCCGCTGTGGGATTCTGGAAGG
glpD-N-R	GCGGATCCACGATCCTCCGTTGTCATCC
glpD-C-F	GCGAATTCTTCGATACACCTGATGGCCG
glpD-C-R	GCGGATCCTTATTGCTCAAGCGGTACGAC
pMut-glpD-F	GTCAAGCTTGTCTTGAAAGAGATCGCATGC
pMut-glpD-R	GCGGATCCGATGTTCCCGCCGCAAAGTC
pAP-plsX-F	GCGGATCCCATTCATAAAGGTGGAGCATGC
pAP-plsX-R	GCGAATTCACCTCCAGACTACTCATCTG
plsX-F1	GGTGTTCAGCCAGAAACAATCG
plsX-R1	GATATGCCTCCTAAATTTTTATCTAAAGTGGCATGCTCCACCTTTATGAATG
plsX-F2	GATAGGCCTAATGACTGGCTTTTATAAACAGATGAGTAGTCTGGAGG
plsX-R2	ACGGACCGCTTACTTCAAGC
cat-F-p	CACTTTAGATAAAAATTTAGGAGGCATATC
cat-R-t	TTATAAAAGCCAGTCATTAGGCCTATC
plsC-F1	ATTACGACAAAGGAAGTGCG
plsC-CΔ7-R1	GTTATCCGCTCACAATTCTTATAGTTCTTTAATCCGCTGATTCAAAG
plsC-F2	GAGATAATGCCGACTGTACTGGCGTTCTGAGAGATTGTCC
plsC-R2	GATGGACATCATTCAGATTG
plsC-cat-F	TAAGAATTGTGAGCGGATAAC
plsC-cat-R	AGTACAGTCGGCATTATCTC
**Construction of expression plasmids for *E. coli***	
PLSB-F	GCGAATTCTTCCAGGAATAGCCAGCAGC
PLSB-R	GCCTGCAGCTGATTTACCCTTCGCCCTG
ECPLSX-F	GCGGATCCGGATTTTCCCCAGGCAACTG
ECPLSX-R	GTCAAGCTTCCTGCTACCGCAGAGTTCCG
YGIH-F	GCGAATTCCGATGGTCTGTTCCCAGTCG
YGIH-R	GCCTGCAGCCAGAAATCACTCGGGATCC
BSPLSX-F	GCGAATTCCATTCATAAAGGTGGAGCATGC
BSPLSX-R	GCGGATCCACCTCCAGACTACTCATCTG
YNES-F	GCGAATTCCAGGAGAAATCAAAATATGATGCATG
YNES-R	GCGGATCCGCTTATAACCATTTTACTTTAGGTTCTG
PLSB-p-F	GCGAATTCGAAACATGAGCGGATACCAC
PLSB-p-R	GCGCATGCTTACCCTTCGCCCTGCGTCGCAC
Spec-F-AatII	GCGACGTCCGAGTTCAAAAATTATATGGAGATCTG
Spec-R-Eam1105I	GCGACTCCCCGTCAGCCTAATTGAGAGAAGTTTCTATAG
TesA-FEcoRI	GCGAATTCTGGTCTCGCCACGTTTGACAAC
TesA-RBamHI	GCGGATCCTCTCCGTTGCTTTATGAGTCATG

Table S4 Oligonucleotides used in this study (continued)

Oligonucleotides	Sequence (5' to 3')
**Gene disruption in *E. coli***	
plsBdelup	CCGTTAAATCCGATGATTCGGGTTTGCGTAGCGCCGCGCGAAACATGAGCGGATACCACAGAATTTCCCATGATTCCGGGGATCCGTCGACC
plsBdeldown	CAGATTATGGAAGGCCGGATAAGGCGTTTTCGCCGCATCCGGCAATTCTCTGATTACCCTTGTGTAGGCTGGAGCTGCTTC
plsXdelup	GGCAACTGGGGAAAGACCAAACCGGGCGGCGACGATACCTTGACACGTCTAACCCTGGCGTTAGGTGTCATGATTCCGGGGATCCGTCGACC
plsXdeldown	TTATATACCGTCACTTGCAAACTGCGAGTTCGCTGGCAGCGTCCTGCTACCGCAGAGTTCCGCTTTTGCCAGGTGTAGGCTGGAGCTGCTTC
ygiHdelup	CGGCGAAATGTGCGTATTATCCACAGATTCATCGTTGTTGAACACGAATTTTCAAAACGGAACAGCTTATGATTCCGGGGATCCGTCGACC
ygiHdeldown	CAGCAACGGCTGGGCAAGCCCGATGAAGGCTATCAGGTCATGTAGATCCACCAGAAATCACTCGGGATCCTGTGTAGGCTGGAGCTGCTTC
**Confirmation of correct gene replacement in *E. coli***	
plsB-check-F	TTAACGCTTTCATTCCGCCC
plsB-check-R	GCCTGCAGAGGCCTACACAACACATCGC
plsX-check-F	CGGAATTCGCCGCAAGGTCATCGCTAAG
plsX-check-R	GCGGATCCACCGTCACTTGCAAACTGCG
ygiH-check-F	CGGAATTCCCGAAAGTTGCTCTATAAATAC
ygiH-check-R	GCAGATCTCCGATGAAGGCTATCAGGTC
k1	CAGTCATAGCCGAATAGCCT
k2	CGGTGCCCTGAATGAACTGC
**Construction of *E. coli* strains harboring IAA-inducible genes**	
Ptrp-FMunI	GCCAATTGGCCGACATCATAACGGTTCTGG
Ptrp-REcoRI	GCGAATTCTGTTCAAAAAAAAGCCCGCTCATTAG
PLSB-F2	GCGAATTCCCAGAGGCTTTACATCGTTTATGTC
PLSB-R2	GCAGATCTTTACCCTTCGCCCTGCGTCGCACTCTC
ECPLSX-F2	GCGGATCCGGATTTTCCCCAGGCAACTG
EC-PLSX-R2	GCGGATCCCCTGCTACCGCAGAGTTCCG
YGIH-F2	GCGAATTCCAAAACGGAACAGCTTATGAGTGC
YGIH-R2	GCAGATCTCCAGAAATCACTCGGGATCC
